# Predictors of Poor Neonatal Outcomes among Pregnant Women in Indonesia: A Systematic Review and Meta-Analysis

**DOI:** 10.3390/nu14183740

**Published:** 2022-09-10

**Authors:** Siti Helmyati, Maria Wigati, Muhammad Hafizh Hariawan, Erri Larene Safika, Mira Dewi, Cindra Tri Yuniar, Trias Mahmudiono

**Affiliations:** 1 Department of Nutrition and Health, Faculty of Medicine, Public Health and Nursing, Universitas Gadjah Mada, Yogyakarta 55281, Indonesia; 2 Center for Health and Human Nutrition, Faculty of Medicine, Public Health and Nursing, Universitas Gadjah Mada, Yogyakarta 55281, Indonesia; 3 Nutrition Study Program, Faculty of Health Sciences, Universitas ‘Aisyiyah Yogyakarta, Yogyakarta 55592, Indonesia; 4 Faculty of Public Health, Mulawarman University, Samarinda 75242, Indonesia; 5 Department of Community Nutrition, Faculty of Human Ecology, IPB University, Bogor 16680, Indonesia; 6 Department of Pharmacology and Clinical Pharmacy, School of Pharmacy, Institut Teknologi Bandung, Bandung 40132, Indonesia; 7 Department of Nutrition, Faculty of Public Health, Universitas Airlangga, Surabaya 60115, Indonesia

**Keywords:** child mortality, health behavior, low birth weight, pregnancy outcomes, prenatal care, smoking

## Abstract

Objectives: This study aimed to examine the association between maternal health behaviors and neonatal outcomes among the Indonesian population. Methods: Articles were collected from PubMed, EBSCO, ProQuest, DOAJ, and GARUDA. Funnel plots and Egger’s tests analyzed indications of publication bias. A Mantel–Haenszel random-effects model was used to see the overall effect size of exposures on outcomes. Heterogeneity was seen based on *I*^2^. Data collected from articles included the author, year of publication, location of the study, study design, number of samples, risk factors, and effect sizes. Results: We identified 24 relevant studies, including eight from the primary databases and 16 from an additional database. A total of 12 studies were included in the meta-analysis, examining the association between maternal health behaviors and neonatal outcomes. The pooled odds ratio (OR) for passive smoking and low-birth-weight (LBW) was 3.41 (95% CI: 1.75–6.63, *I*^2^ = 40%, four studies). The pooled OR for incomplete antenatal care (ANC) and LBW was 6.29 (95% CI: 2.11–18.82, *I*^2^ = 70%, four studies). The pooled OR for incomplete ANC and neonatal mortality was 2.59 (95% CI: 1.01–6.66, *I*^2^ = 93%, four studies). Conclusions: The results indicated that pregnant women with incomplete ANC had a higher risk of LBW and neonatal mortality, and those who were passively exposed to smoking had a higher risk of LBW. Further investigations are needed, considering the high heterogeneity found, and additional meta-analyses should be based on the variations of socio-demographic conditions.

## 1. Introduction

Maternal and child health and nutrition (MCHN) remain a problem in Indonesia and many low- and middle-income countries, which might as well burden the economy of a country’s health system [1]. Compared to neighborhood countries in Southeast Asia, protection for Indonesian young children under five is still needed to lower the mortality rate among them [2]. The country’s latest survey in 2015 documented the maternal mortality ratio of 305 per 100,000 live births [3]. According to routine national health data, the aggregate number of maternal deaths increased from 4197 in 2019 to 4627 in 2020. Additionally, it was reported that 28,158 children under five died in 2020. Of that number, 72% were neonates [4].

It was said that to pursue the optimum potential of the children, physical and social environmental balance was necessary. The domain covered individual levels such as nurturing culture within family, peers, and school conditions, to the community at a larger system [5]. However, the period between pregnancy and children of 24 months old, also known as the first 1000 days of life of children, might be the most prominent factor [6,7]. That being said, maternal diet and lifestyle affect neonatal outcomes. For example, poor antenatal care, environmental exposure, chronic malnutrition, inadequate personal hygiene and sanitation, anemia in pregnant women, and high second-hand smoke exposure during pregnancy contribute to poorer health outcomes for children such as stunting, low birth weight (LBW), impaired cognitive function, altered metabolic dynamics, and lower immune system response of the children [8].

The issue of MCHN has been a concern for many practitioners for years [9,10,11]. Nevertheless, information from Indonesian studies was lacking. It is unfortunate since as the largest archipelago country in the world with more than 270 million people in 2022 [12], diverse cultures and demographic situations might affect maternal behaviors that influence neonatal outcomes. Several examples include the effect of socio-culture on the perceived concept of health, traditional care during pregnancy, and health-seeking behaviors within community groups [13,14,15,16,17,18,19,20,21,22,23,24]. These situations might be Indonesia-specific and different from other cultures across countries, which could be an interesting case study for public health stakeholders worldwide. Thus, we aimed to elaborate on various Indonesian studies published in domestic and international sources. The results are expected to support evidence-based policy making in Indonesia and other countries with similar characteristics.

The findings in this study should be considered with some limitations. First, several articles included in this meta-analysis had a considerable risk of bias, with a NOS score below five. Second, there were high scores of heterogeneities for the meta-analysis of (1) incomplete ANC and LBW and (2) incomplete ANC and neonatal mortality. Third, funnel plots are disavowed with such a small sample size (*n* < 10 studies), as the inference of the plot itself can be biased.

## 2. Materials and Methods

### 2.1. Study Design and Research Sample

This systematic review and meta-analysis followed the guidelines set by the Preferred Reporting Items for Systematic Reviews and Meta-Analyses (PRISMA) Statement [25]. The research sample in this meta-analysis included articles published from January 1990 to May 2021, which were contained in the PubMed, EBSCO, ProQuest, and DOAJ databases. We were specifically looking at the impact of health-related behavior of pregnant women on neonatal outcomes.

### 2.2. Operational Definition

The exposure or case was any health-related behaviors of Indonesian pregnant women, such as supplement consumption, exposure to smoke, and pregnancy care. Meanwhile, neonatal outcomes were defined as the health conditions of newborns until the age of 28 days. It included birth weight, birth length, death, and other conditions. For analysis, LBW was defined as a baby born with a weight below 2500 g, while neonatal mortality or neonatal death was defined as the death after a live birth during the first to 28 days of life [26].

### 2.3. Research Query

Queries used in this research were adjusted to MeSH. We search from PUBMED, PROQUEST, DOAJ, and EBSCO as primary databases and GARUDA as the additional database with the following query “pregnant women” AND (“smoking behavior” OR “secondhand smoking” OR “passive smoking” OR “dietary supplements” OR “snack” OR “snack food” OR “snacking” OR “dietary fiber” OR “dietary calcium” OR “antenatal care” OR “prenatal care” OR “sleeping habit” OR “sleep” OR “dietary sugar” OR “protein deficiency” OR “dietary energy intake” OR “dietary protein intake” OR “protein-energy malnutrition” OR “dietary sodium chloride OR hygiene OR activities”) AND (“body length” OR “birth weight” OR “low birth weight” OR “neonatal anemia” OR “pregnancy outcome” OR “head circumference” OR “upper arm” OR “upper arm circumference” OR “infant” OR “newborn”) AND “Indonesia”.

### 2.4. Additional Resource

We added additional journals from GARUDA [27], a scientific article database managed by Indonesia’s Ministry of Education, Culture, Research, and Technology. It contains articles either in Bahasa Indonesia or English from local researchers. GARUDA is a comprehensive database covering all subjects from arts and humanities, behavioral sciences, and social sciences to medicine, pharmacology, physical sciences, engineering, and mathematics. It is designed for browsing, indexing, abstracting, monitoring, and improving the standard of scholarly publications in Indonesia. To date, it contains more than 2500 publishers and 13,000 journals.

### 2.5. Data Collection Procedures

Articles were selected based on population–exposure–comparison–outcome (PECO). The population in this study included pregnant women in Indonesia. The exposure was health-related behavior of pregnant women. The comparison for this study was pregnant women in Indonesia without direction. Meanwhile, pregnancy outcomes such as birth weight, birth length, death, and other conditions in newborns were measured as outcomes.

**Figure 1:**
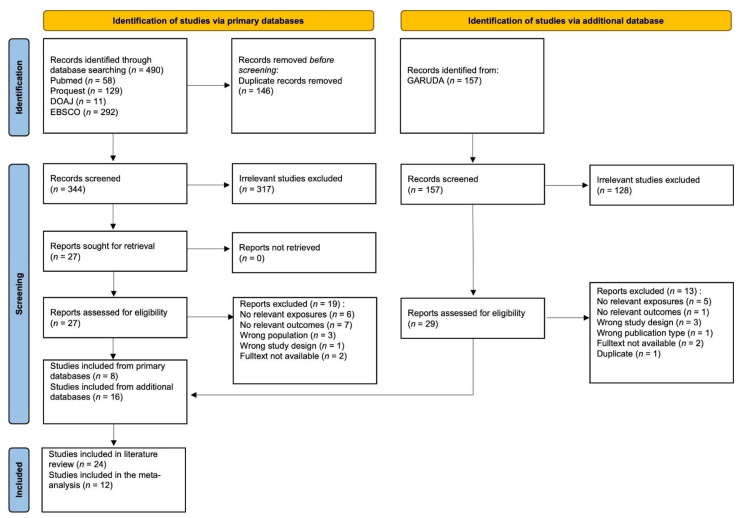
Data collection process.

The data collection process can be seen in Figure 1. We collected full-text original articles from January 1990 to May 2021. The inclusion criteria for the articles were: (1) original articles in Bahasa Indonesia or English, (2) original research articles fulfilling PECO criteria, and (3) observational studies conducted in Indonesia, either cohort or case-control. Articles were excluded if full texts were not available or data incomplete. After collecting the articles, three reviewers (MW, ELS, MHH) who are fluent in Bahasa Indonesian and English separately examined the title, the abstract, and the full text. We moved to the next stage of assessment when the consensus was achieved in the initial review phase. While checking duplicates and reviewing articles, we used Rayyan, a web and mobile app for systematic reviews [28]. We tabled the articles’ information, including the authors’ names, year of publication, study location, study design, sample size, risk factors, and effect size.

### 2.6. Data Analysis

Review Manager 5.310 and STATA 1611 were used for the data analysis to analyze the pooled effect size, including odds ratio (OR) and heterogeneity from the collected articles. The Mantel–Haenszel random-effects method was used to conduct the meta-analysis. A forest plot was produced to show the estimated effect, the number of variations between studies, and confidence intervals (CI) from individual studies meta-analyses [29]. For articles that have an effect size of relative risk (RR), calculations in Stata 16 were performed to obtain the effect size in the form of odds ratio (OR) formulated from summary data such as effect size and CI to convert RR to OR before analyzing the pooled effect size. Substantial heterogeneity was based on the *I^2^* value (*I*^2^ = 50–90%) [30]. Analyses of funnel plots and Egger’s test were conducted to see potential publication bias. The Newcastle–Ottawa Scale (NOS) was used to assess the quality of the selected articles [31]. Due to the limited research in Indonesia, specifically for articles in Indonesian, we used SINTA [32], a Science and Technology Index developed by the Ministry of Research and Technology of Indonesia, to assess the quality of articles in addition to using NOS. We were only looking for journals with a SINTA index 1–3. Journal assessment at SINTA was based on several aspects, such as the number of articles with the Scopus index, the number of citations on Scopus, and the number of sources on Google Scholar.

## 3. Results

### 3.1. Characteristics of Included Studies

This study retrieved 490 articles, with 59 articles from PUBMED, 129 from PROQUEST, 11 from DOAJ, and 292 from EBSCO. After duplications were removed, 344 articles were screened for abstracts, which resulted in 27 articles screened for full-text review. As many as eight articles were selected for this study. Additional resources were also included from GARUDA, an Indonesian scientific journal database. Using the same query, we found 157 journal articles, with 16 papers selected from full-text reviews. Finally, 24 articles from additional sources were selected, and 12 were analyzed for meta-analysis. Twelve studies were not included in the meta-analysis because they did not have comparable outcomes or interventions. There should be at least two studies with the same outcome for a meta-analysis to be conducted [33].

Table 1 shows all studies included in the literature review. All studies included were conducted across Indonesia. As for studies in the Indonesia database, we only had those published in journals with SINTA index three or above. Six studies were found on Sumatera Island [34,35,36,37,38,39], five on Java Island [40,41,42,43,44], four on Sulawesi Island [45,46,47,48], two on Borneo Island [49,50], three in East Nusa Tenggara [51,52,53], one in Bali [54], and one in Maluku Island [55]. Seventeen studies were conducted by case-control study design, and eight by cohort study design.

Regarding the year of publication, all studies were published between 2010 and 2020. Maternal health behaviors identified from the collected studies based on the queries inputted were ANC visits, active and passive smoking, iron and folic acid supplementation, sun exposure, obedience to traditional pregnancy care, fasting, and the use of household pesticides, and macronutrient intake. In contrast, the pregnancy outcomes were low birth weight, neonatal mortality, stillbirth, head circumference, and pneumonia in the first postnatal year. However, only a few maternal health behaviors and outcomes were eligible to be analyzed for meta-analysis. The exposures for this meta-analysis were maternal health behaviors such as active and passive smoking and ANC visits, while the outcomes were LBW and neonatal mortality.

### 3.2. Quality of Studies

The NOS assessment form was used for assessing the quality of nonrandomized studies, including case-control and cohort studies. Sixteen studies included in this systematic review and meta-analysis had scores above or equal to 5 points, which means they had no considerable risk of bias. Approximately eight studies scored below 5 points, regarded as having a potential bias [56]. Complete results of the quality analysis for all articles can be seen in Table 1.

Relation between maternal smoking behaviors, antenatal care, and infants born with low birth weight.

A meta-analysis examining the relationship between smoke exposure and LBW was conducted on four studies discussing the association between maternal smoke exposure and birth weight. Those research studies were performed in western Indonesia (Lampung), middle (Bali, Kalimantan), and Eastern Indonesia (Sulawesi). Three studies used case control, and one used retrospective cohort design. From the case-control studies, there were 143 cases of infants with LBW and 143 controls, while in the cohort study, there were 45 subjects exposed to SHS and 22 subjects not exposed to SHS.

Another four studies were included to assess the association between ANC and LBW. These studies used a case-control design and were conducted in western Indonesia. In total, there were 138 cases of mothers who gave birth to a LBW baby and 220 mothers as controls who gave birth to average birth weight (NBW) baby.

**Figure 2:**
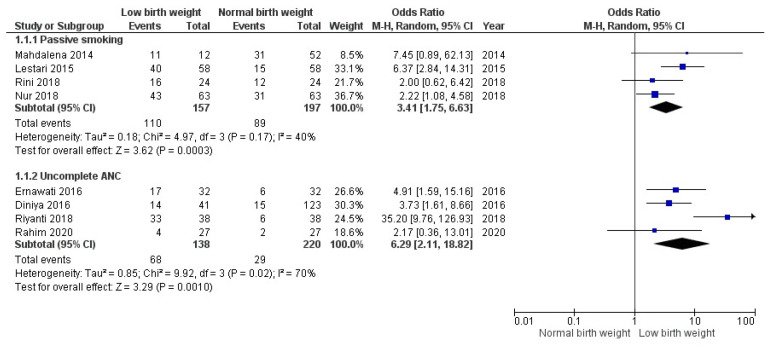
Association between passive smoking, active smoking, incomplete ANC, and LBW.

In Figure 2, exposures were analyzed to see the overall effect on the behavior of pregnant women toward LBW babies. Based on the analysis of pooled articles, the odds of babies born with LBW were 6.3 times greater in pregnant women with incomplete ANC (95% CI: 2.11–18.82) and 3.4 times greater in pregnant women who became passive smokers (95% CI: 1.75–6.63). Although the overall effect size for incomplete ANC was greater than passive smoking, there was an indication of a high variation in the research on the relationship between incomplete ANC and LBW cases in Indonesia, as indicated from *I*^2^ > 50% and *p*_heterogeneity_ < 0.05.

### 3.3. Relation between Antenatal Care and Neonatal Mortality

**Figure 3:**
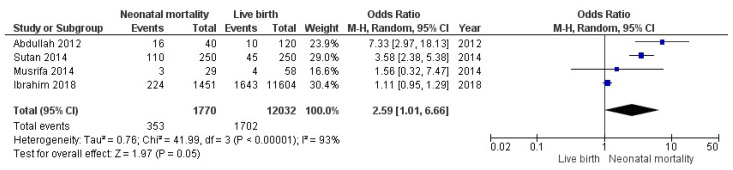
Association between incomplete ANC and neonatal mortality.

Four studies from 2012 to 2018 were analyzed to understand the relationship between incomplete ANC and neonatal mortality. Three studies were case control, and one was a cohort. From the case-control studies, there were 319 cases of mothers with a history of neonatal mortality and 428 controls. Meanwhile, the cohort study found 1867 subjects exposed to low ANC and 11,118 subjects with good ANC. Figure 3 shows that the odds of neonatal mortality were increased 2.5 times if the mother did not obtain complete ANC (95% CI: 1.01–6.66). However, this result must be carefully examined since the heterogeneity test showed 91.53%. There was a wide range of sample sizes between Ibrahim et al. [55] and the other three studies, which may contribute to the results.

### 3.4. Potential for Publication Bias Analysis

Considering the potential for publication bias in our results, we conducted Egger’s tests followed by a funnel plot analysis. The Egger’s tests in Table 2 showed that the *p* values of all pairs of maternal health-related behaviors and outcomes were >0.05, indicating no publication bias. However, due to the small number of studies included in this meta-analysis, the power of Egger’s test to detect publication bias is low.

**Figure 4:**
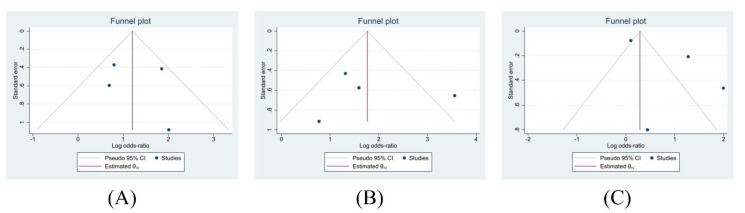
Funnel plot graph: (**A**) passive smoking and LBW, (**B**) incomplete ANC and LBW, and (**C**) incomplete ANC and neonatal death.

Corresponding with the results of Egger’s tests, the funnel plots in Figure 4 demonstrate the variety of shapes of the pooled studies. Figure 4B,C seemed asymmetrical, while Figure 4A seemed more symmetrical. This pattern supported previous results that showed that the studies had a high publication heterogeneity. Therefore, the conclusion was difficult to reach since the number of studies was limited.

## 4. Discussion

This systematic review and meta-analysis evaluated the association between maternal health behaviors and neonatal outcomes in Indonesia. These findings confirmed that mothers who did not have ≥4 ANC visits had a higher risk of giving birth to LBW infants (OR 6.29, 95% CI: 2.11–18.82), as did mothers who were exposed to cigarette smoke (OR 3.41, 95% CI: 1.75–6.63).

Studies from developing countries have shown evidence about the importance of ANC in mitigating the prevalence of LBW and neonatal mortality [44]. A study in Sulawesi, Indonesia, found that pregnant women who were not receiving several ANC contents, including regular checks of blood pressure, hemoglobin, and body weight measurement, had a higher risk of giving birth to LBW infants [47].

The WHO recommended the Focused Antenatal Care (FANC) model, indicating that pregnant women should make at least four ANC visits. This has been updated to 2016 WHO ANC Model, which recommends a minimum of eight ANC contacts during pregnancy [57]. It aims to decrease maternal morbidity and mortality. A survival analysis from the IDHS reported that ANC, not postnatal care, reduced the risk of neonatal death by 51%. Unfortunately, several studies found disparities in ANC utilization in developing countries, including Indonesia. Discrepancies could occur due to geographical, demographic, socioeconomic conditions, and cultural differences, resulting in decreased access, service quality, and affordability [58].

A previous study reported that the eastern part of Indonesia had the lowest distribution of ≥4 ANC visits. Its proportion was centered in the central region, namely the Java-Bali region, followed by the western part. Regional geographic conditions in eastern Indonesia are also more extreme and challenging to reach than in their western counterparts, making some areas fall into a small category. Further, Java-Bali and Kalimantan regions were dominated by wealthier women, while the remaining sites were by the poor ones [52]. Socio-economic factors were also related to ANC visits among adolescents and young women in Indonesia [59]. To overcome this disparity, the Indonesian government launched the world’s most comprehensive single-payer health insurance called Jaminan Kesehatan Nasional (JKN) to ensure the nation’s universal health coverage [60]. This expansion of health insurance coverage has improved access to MCHN services, which is helpful for the poorest quantile households and remote areas in the eastern part of Indonesia. However, the discrepancy in maternal health services coverage persists across socioeconomic groups and geographical regions, which may be caused by the inequitable distribution of the subsidy and maternal health services provided by JKN [61].

Our study found that passive smoking was associated with a higher risk of LBW in Indonesia. Exposure to tobacco smoke (ETS), or passive smoking, is passive second-hand smoke (SHS) exposure to tobacco smoke from an active smoker and has harmful impacts on health and pregnancy outcomes. Following our results, other studies have shown increased risks of LBW, preterm delivery, and trimmer head circumference in mothers exposed to SHS [62]. A meta-analysis by Leonardi-Bee et al. [63] found that ETS reduces mean birth weight by 33 g or more and increases LBW by 22%. Although other studies supported our results, we found heterogeneity in our analysis that socio-demographic variations might cause. Two studies were conducted in hospital settings [47,50], one in a maternity clinic [36], and the other in a community setting [54]. The sample size in all studies was relatively small, with a wide range of CI.

A study in Ambon, Indonesia, reported that pregnant passive smoker women had a 2.8-times higher chance of stillbirth after adjusting for ANC quality, anemia, and education [55]. Tobacco smoke contains many harmful toxins and may adversely affect pregnant women through various mechanisms. Research showed that passive smokers possibly inhale fetal toxins in greater concentrations. Several mechanisms have been suggested concerning why passive smoking affects neonatal birth weight. Nicotine or polycyclic aromatic hydrocarbons from cigarette smoke may directly affect fetal growth, with nicotine constricting uteroplacental arteries while reducing blood flow and oxygen to the fetus and other toxins that may have unidentified mechanisms [63].

We found additional factors that may influence adverse neonatal outcomes in Indonesia. A study in East Nusa Tenggara, Indonesia, found that pregnant mothers chose not to conduct standard ANC, kept working hard in domestic work, and followed several food restrictions because of the cultural beliefs that follow ‘traditional pregnancy care’ to ensure a smooth delivery. In low socio-economic conditions, smooth pregnancy and delivery are considered valuable and could save more costs. Further, these recommendations are passed down by parents-in-laws [51]. These ‘traditional pregnancy care’ practices must be carefully considered in MCHN programs since many cultures in Indonesia also have some rules and taboos concerning pregnant women. The majority are afraid and uncomfortable disobeying the traditional rules [64].

A study in Padang, Indonesia, reported that pregnant women with inadequate energy, protein, fat, and carbohydrate intake had a higher risk for LBW [39]. A study in Indonesia said that fasting pregnant women had a significantly lower energy intake during the one month of Ramadhan, but fasting was not associated with LBW [42]. This result was similar to studies in other countries [65]. Acute respiratory infection (ARI), pneumonia, and vitamin D deficiency at birth are common in Indonesia. A cohort study in Yogyakarta, Indonesia, reported that vitamin D supplementation and safe sun exposure during pregnancy had the potential to reduce ARI incidence in infants [41]. Therefore, appropriate macro- and micronutrient intake plays a role in protecting against adverse neonatal outcomes and should be emphasized during pregnancy.

Findings in our study suggest that local governments should increase the ANC quality in each district, along with the JKN intervention in providing health insurance for pregnant mothers and equal distribution of maternal health services, to narrow the gap in maternal health services across the country. Health providers must have strong communication skills and knowledge of nutrition and health behavior for pregnant women, so that they can effectively give proper education based on local customs. Further, health providers and the government need to cooperate with local organizations to campaign for smoking cessation programs and strengthen education about the dangers of cigarette smoke for pregnant women and infants.

The findings in this study should be considered with caution. First, several articles included in this meta-analysis had a considerable risk of bias, with a NOS score below five. Second, there were high scores of heterogeneities for the meta-analysis of (1) incomplete ANC and LBW and (2) incomplete ANC and neonatal mortality. Third, funnel plots are disavowed with such a small sample size (*n* < 10 studies), as the inference of the plot itself can be biased. As proposed by Sterne et al. [66], it would be hard to achieve any definite conclusions within a meta-analysis with high heterogeneity since there were possible socio-demographic variations and seasonality effects in those studies. This is not an exaggeration considering the situation of Indonesia as a vast archipelago country. Although these limitations impede concluding a fixed relationship between maternal health behaviors and neonatal outcomes in Indonesia, this analysis still provides broadening views and improves the scarcity in the literature. These findings also show the need to improve the quality of the research and publications conducted in Indonesia. Furthermore, the authors would like to propose the importance of the socio-demographic and cultural approach analysis based on the study sites in Indonesia. Study results might be different because of the high variabilities of these factors.

## 5. Conclusions

Our meta-analysis of studies in Indonesia found that pregnant women who had less than four ANC visits had a higher risk of giving birth to LBW infants. Pregnant women exposed to cigarette smoke also had a higher chance of LBW. These results must be carefully interpreted since we discovered high heterogeneity among the included studies. Further investigation needs to be performed to assess the health-related behaviors among pregnant women and their association with neonatal outcomes in Indonesia. The evidence is still scarce, and we can reach better conclusions about the significant associations with more precise findings.

## Figures and Tables

**Table 1:** General characteristics of studies.

No	First Author, Publication Year	Study Year	Study Design	Location	Study Subject		Control		Health-Related Behaviors	Neonatal Outcomes	Effect Size	95% CI	Other Results	Article Quality ^a^
1	Abdullah et al., 2012 [45]	2009	Case-control	Makassar, South Sulawesi	40	Mothers with early neonatal death history	120	Mothers without early neonatal death history	0–4 ANC visits	Early neonatal death	OR 1.558	0.32–7.47	*p* value = 0.000; B = 0.748; Sig = 0.650	7
									0–3 ANC visits	Early neonatal mortality	OR 7.3	N/A		
2	Abdullah et al., 2016 [52]	2013	Matched case-control	East Nusa Tenggara	152	Mothers with neonatal death history reported to health service	308	Mothers without neonatal deaths history reported to health service	Did not take all recommended iron supplements	Neonatal death	OR 2.27	1.13–4.54	Several infants’ habits also significantly influenced the risk of neonatal death (did not initiate early breastfeeding, did not practice the kangaroo method and had health problems)	7
3	Alfianti and Darmawati, 2016 [34]	2016	Retrospective	Banda Aceh	35	Mothers who gave birth to LBW baby	N/A	N/A	Smoking	Low-birth weight	N/A	N/A	Results were descriptively analyzed	1
4	Anggrahini et al., 2020 [51]	2016–2017	Case-control	East Nusa Tenggara	50	Mothers who gave birth to LBW baby	50	Mothers who gave birth to NBW baby	Obedience to traditional pregnancy care	Low-birth weight	N/A	N/A	Path analysis found that LBW was significantly affected by maternal health status and obedience to the traditional pregnancy rate	5
5	Astuti, 2020 [35]	2020	Case-control	South Bengkulu	15	Mothers who gave birth to LBW baby	15	Mothers who gave birth to NBW baby	Have complete ANC visits	Low-birth weight	8	N/A	The author did not define what complete ANC visits is	3
6	Diniya et al., 2016 [49]	2013–2015	Case-control	Banjar	41	Mothers who gave birth to LBW baby	123	Mothers who gave birth to NBW baby	Have complete ANC visits	Low-birth weight	OR 3.73	1.61–8.66	ANC visits were the only health behavior discussed in this article	4
7	Ernawati, 2016 [40]	N/A	Case-control	Pati, Central Java	32	Mothers who gave birth to LBW baby	32	Mothers who gave birth to NBW baby	Frequency ANC visits	Low-birth weight	N/A	N/A	-	7
									Have complete ANC visits	Low-birth weight	OR 4.911	1.591–15.157		
8	Heldawati et al., 2018 [46]	2015–2016	Retrospective cohort	Palu, Central Sulawesi	34	Preeclamptic pregnant women	34	non-preeclamptic pregnant women	<4 ANC visits	Low-birth weight	*p*=0.013	N/A	-	5
9	Ibrahim et al., 2012 [55]	2006–2007	Cohort	Indonesia	1867	Pregnant women with 2–3 ANC visits during pregnancy	11,118	Pregnant women with four or more ANC visits during pregnancy	4–6 ANC visits	Neonatal mortality	RR 1.01	0.61–1.68	The frequency of ANC visits in the third trimester is significantly associated with the risk of neonatal death.	7
									7–9 ANC visits	Neonatal mortality	RR 0.65	0.40–1.08		
									Ten or more ANC visits	Neonatal mortality	RR 0.60	0.32–1.13		
10	Lestari et al., 2015 [54]	2013	case-control	Gianyar, Bali	58	Infants with low birth weight	58	Infants with average birth weight	Husband’s smoking exposure	Low-birth weight	OR 6.37	2.836–14.309	-	3
									Family members smoking exposure	Low-birth weight	OR 6.577	2.894–14.948		
11	Mahdalena, 2014 [50]	2012	Retrospective cohort	South Kalimantan	45	Active and passive smoking pregnant women	22	Nonsmoking pregnant women	Non-smoking	Low-birth weight	*p* = 0.78	N/A	-	3
									Passive smoking	Low-birth weight				
									Active smoking	Low birth weight				
12	Musrifa et al., 2014 [53]	2013	Case-control	East Nusa Tenggara	29	Mothers with early neonatal death history	58	Mothers without early neonatal death history	Husband’s smoking exposure (passive smoking)	Early neonatal death	OR 2.758	0.72–10.50	-	6
13	Nur et al., 2016 [47]	2015	Case-control	Palu, Central Sulawesi	58	Mothers giving birth to LBW infant	116	Mother giving birth to normal weight infant	Body weighing	Low birth weight	OR 2.5	1.26–5.03	The pregnancy gap was found to be another risk factor for LBW	4
									Blood pressure checking	Low birth weight	OR 2.69	1.39–5.18		
									Hemoglobin level examination	Low birth weight	OR 3.15	1.45–6.85		
14	Nur, 2018 [48]	N/A	Case-control	Palu, Central Sulawesi	63	Mothers giving birth to LBW infant	63	Mother giving birth to normal weight infant	Passive smoking	Low birth weight	OR 2.21	1.07–4.58	Premature rupture of membranes and light placenta is found to be the other risk factors of LBW	3
15	Oktaria et al., 2021 [41]	2015–2017	Community-based cohort	Yogyakarta	422	Pregnant women and their infants from birth until 12 months	N/A	N/A	Mother sun exposure for the entire pregnancy (weekday)					8
									Q2 v Q1	pneumonia onset in the first postnatal year	HR 0.72	0.41–1.26		
									Q3 v Q1	pneumonia onset in the first postnatal year	HR 0.85	0.49–1.49		
									Q4 v Q1	pneumonia onset in the first postnatal year	HR 0.25	0.25–0.90		
									Mother sun exposure for the entire pregnancy (weekend)					
									Q2 v Q1	pneumonia onset in the first postnatal year	HR 0.68	0.38–1.22		
									Q3 v Q1	pneumonia onset in the first postnatal year	HR 0.91	0.53–1.55		
									Q4 v Q1	pneumonia onset in the first postnatal year	HR 0.46	0.24–0.87		
									Smoke exposure					
									Father smokes every day	pneumonia onset in the first postnatal year	HR 1.57	1.02–2.41		
16	Paunno et al., 2015 [55]	2007–2008	Case-control	Ambon, Maluku	69	Mothers with stillbirth history	69	Mothers without stillbirth history	Passive smoking	Stillbirth	OR 3.36	1.22–10.17	The other risk factors of stillbirth were ANC quality and anemia status.	6
17	Rahim, 2020 [44]	2017–2018	Case-control	Kuningan, West Java	27	Mothers giving birth to LBW infant	27	Mother giving birth to normal weight infant	0–3 ANC visits	Low birth weight	OR 2.17	0.36–13.01	The small number of subjects could be the cause of the insignificant results	8
18	Rini et al., 2018 [36]	2012–2015	Case-control	Lampung	22	Mothers giving birth to LBW infant	22	Mother giving birth to normal weight infant	Passive smoking	Low birth weight	OR 2	0.62–6.42	Passive smoking is the only health behavior analyzed	4
19	Riyanti et al., 2018 [37]	2015–2016	Case-control	Bener Meriah, Aceh	38	Mother giving birth to LBW infant	38	Mother giving birth to normal weight infant	Active Smoking	Low birth weight	OR 1.45	0.43–1.02	Maternal age, number of children, and anemia are also found to be the risk factors for LBW	5
									Four or more ANC visits	Low birth weight	OR 0.28	0.08–1.02		
20	Savitri et al., 2018 [42]	2012–2014	Cohort	Jakarta	1099	Pregnant women Ramadhan exposure	252	Pregnant women without Ramadhan exposure	Fasted at any trimester	birth weight	β −63.7	−230.5 to 103		8
									Fasted in the first trimester	birth weight	β −23.4	−246.8 to 200.1		
									Fasted in the second trimester	birth weight	β −35.3	−224.2 to 153.7		
									Fasted in the third trimester	birth weight	β −121.8	−318 to 74.4		
21	Soesanti et al., 2020 [43]	2012–2017	Cohort	Jakarta	133	Pregnant women who use household pesticide	151	Pregnant women who did not use household pesticide	Use of household pesticide	birth weight	β −121.4	−227.6 to −15.2		7
									Use of household pesticide	birth length	β −4.0	−9.3 to 1.4		
									Use of household pesticide	Head circumference	β −6.4	−12.1 to −0.7		
22	Sutan et al., 2014 [38]	2010–2012	Unmatched case-control retrospective	Aceh	250	Mothers with LBW babies who died during the neonatal period	250	Mothers with LBW babies who survived during the neonatal period	0–3 ANC visits	Neonatal mortality among low birth weight	OR 3.57	2.38–5.38		7
23	Syari et al., 2015 [39]	2015	Case-control	Padang, West Sumatera	19	Mother giving birth to LBW infant	21	Mother giving birth to normal weight infant	Energy intake	Low birth weight	OR 76	7.7–754	Macronutrient intake was the only health behavior discussed in the article	7
									Protein intake	Low birth weight	OR 8.5	1.54–47		
									Fat intake	Low birth weight	OR 7	1.7–30.5		
									Carbohydrate intake	Low birth weight	OR 12	2.7–53.3		
24	Titaley et al., 2010 [24]	1994, 1997, 2002–2003	Cohort	Indonesia	442	Mothers with recent early death neonate	40134	Mothers with singleton live-born infants	Iron/folic acid supplementation	Neonatal mortality	HR 0.53	0.36–0.77	Iron and folic acid supplementation provide the main protective effect combined with no other form of neonatal care or <2 tetanus toxoid injections	7

^a^ Newcastle–Ottawa Scale [35]. N/A, data not available.

**Table 2:** Publication bias analysis.

Risk Factors	Outcomes	*p* Value ^a^
Passive smoking	Low birth weight	0.656
Incomplete ANC	Low birth weight	0.876
Incomplete ANC	Neonatal mortality	0.717

^a^ Egger’s test, significant if *p* < 0.05.

## Data Availability

The dataset can be obtained by contacting the researcher.
